# Kinetic Laser Absorption Spectroscopy of Vibrationally Excited Hydroxyl Radicals on Infrared Transitions ν = 3 ← 1 and ν = 4 ← 2

**DOI:** 10.3390/molecules30030540

**Published:** 2025-01-24

**Authors:** Daria M. Plastinina, Evgeni N. Chesnokov, Pavel V. Koshlyakov, Lev N. Krasnoperov

**Affiliations:** 1 Institute of Chemical Kinetics and Combustion, Siberian Branch of Russian Academy of Sciences, 630090 Novosibirsk, Russia; chesnok@kinetics.nsc.ru (E.N.C.); pvk@kinetics.nsc.ru (P.V.K.); 2 Department of Chemical Physics, Novosibirsk State University, 630090 Novosibirsk, Russia; 3 Department of Chemistry and Environmental Science, New Jersey Institute of Technology, Newark, NJ 07102, USA

**Keywords:** vibrationally excited hydroxyl radical, DFB laser diode, vibrational relaxation, overtone spectroscopy

## Abstract

The kinetics of vibrationally excited OH(ν = 1) and OH(ν = 2) radicals was studied by time-resolved laser absorption in the overtone IR region. Two DFB laser diodes, 1509.3 and 1589 nm, were used. The technique allowed for the reliable study of the vibrational relaxation kinetics as well as the relative populations of the vibrationally excited states. The yields of OH(ν = 1) and OH(ν = 2) in the reaction O(^1^D) + H_2_O were determined. The rate constant of OH(ν = 1) relaxation in collision with water molecules was obtained ((9.2 ± 2.0) × 10^−12^ cm^3^/s). The dynamics of OH(ν = 1) and OH(ν = 2) populations were analyzed in detail, which made it possible to separately determine the relative contribution of the vibrational ladder relaxation channels OH(ν = 2) → OH(ν = 1) → OH(ν = 0) and the direct relaxation OH(ν = 2) → OH(ν = 0).

## 1. Introduction

Vibrationally excited hydroxyl radicals are produced in many reactions important in atmospheric chemistry [1]. The main reactions are the reactions of the electronically excited oxygen atom O(^1^D) with hydrogen-containing compounds such as water, hydrogen, methane, other hydrocarbons, etc.:

O(D1)+H2O →OHv=0,1,2+ OHv=0,1,2;

O(D1)+H2→O+ OHv=0…5;

O(D1)+CH4 →CH3+ OHv=0…4.

Another example of an exothermic reaction producing vibrationally excited hydroxyl radicals is the reaction of a hydrogen atom with nitrogen dioxide:

$$H+{\mathrm{NO}}_{2}\to \mathrm{NO}+\mathrm{OH}\left(v=0\ldots 3\right).$$

Once produced, vibrationally excited hydroxyl radicals either undergo vibrational relaxation or are consumed in further chemical reactions. Reliable evaluation of the role of these processes in the atmosphere requires detailed kinetic data on the yields and the rate constants of these processes.

Laser-Induced Fluorescence, LIF, is the most common method used for kinetic studies of the formation and subsequent reactions of vibrationally excited hydroxyl radicals. The yields of vibrationally excited hydroxyl radicals in the photolysis of H_2_O_2_ and HNO_3_ were measured in [2,3], respectively. The formation of vibrationally excited hydroxyl radicals was studied in the reactions of an O(^1^D) atom with H_2_O [4,5,6,7], CH_4_ [8], and H_2_ [9]. The vibrational relaxation of OH on H_2_O and D_2_O molecules was investigated in [8]. There are studies on vibrationally excited OH relaxation on NO_2_ [10], HNO_3_, DNO_3_ [11], SO_2_ [12,13], O_3_ [14], and acetone [15]. Spectroscopic studies of vibrationally excited states of OH in the equilibrium conditions were also performed using Electron Paramagnetic Resonance [16] and Laser Magnetic Resonance methods [17]. In [18,19], the chemiluminescence of vibrationally excited OH formed in the reaction H + O_3_ was studied. The role of vibrationally excited hydroxyl radicals, HO (ν ≤ 9), in the stratosphere was discussed. Also, the chemiluminescence of vibrationally excited OH in the products of the H + NO_2_ reaction was studied in [20].

It should be noted that although the LIF method is highly sensitive, allowing the low-pressure detection of OH in various states, it requires careful calibrations and consideration of numerous experimental factors for the determination of the absolute OH concentrations or/and relative populations of its vibrational states. On the contrary, detection by absorption in the IR region easily allows reliable data on the absolute concentrations to be obtained, since the parameters of IR spectral lines of OH are well known.

In this work, we demonstrated the detection of OH(ν = 1) and OH(ν = 2) by absorption at the ν = 3 ← 1 and ν = 4 ← 2 transitions using tunable diode lasers. To the best of our knowledge, this is the first use of IR laser diodes for the direct monitoring of OH(ν = 1) and OH(ν = 2). The temporal profiles of the absolute concentrations of OH in the different states, formed in the reaction of O(^1^D) with water molecules, were recorded. The yields of the vibrational states as well as the collisional relaxation rate constant were determined.

## 2. Materials and Methods

### 2.1. Experimental Setup

**Figure 1:**
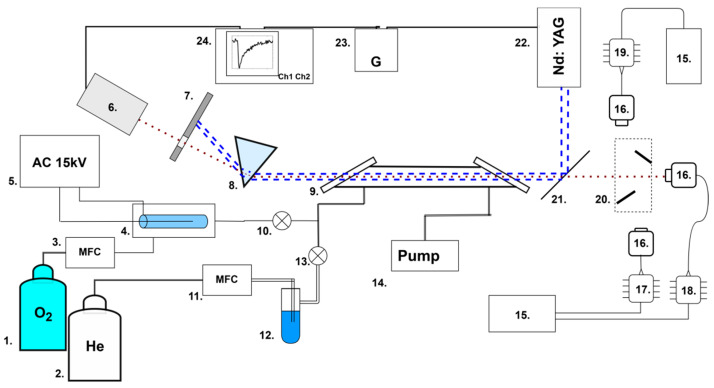
Experimental setup: 1, 2—gas cylinders; 3, 11—flow regulators; 4—ozonator; 5—high-voltage source; 6—photodiode; 7—screen; 8—prism; 9—gas cell (30 cm); 10, 13—valves; 12—water vapor saturator; 14—pump; 15—temperature controller; 16—optical collimator; 17—DFB laser diode (1431 nm); 18—DFB laser diode (1509 nm); 19—DFB laser diode (1590 nm); 20—two-mirror switching system; 21—UV reflective mirror; 22—Nd:YAG UV laser; 23—timing generator; 24—oscilloscope. The red dotted line represents infrared laser radiation; the blue dotted lines are ultraviolet laser radiation.

The experimental setup, described in detail in [21], was modified by adding a mechanical two-mirror switching system 20 between three laser diodes 17, 18, and 19 (Figure 1).

Briefly, the main elements were a flow gas system; three DFB (distributed-feedback) InGaAsP laser diodes 17, 18, and 19 with temperature controllers 15; and a pulsed UV laser 22. The two-mirror switching system 20 was used to switch three IR laser diodes. In the position shown in Figure 1, the right laser (1431 nm) is used. When the mirrors are moved upwards, the beam from the upper laser is directed into the cell. And when the mirrors are moved downwards, the beam of the lower laser is directed into the cell.

Hydroxyl radicals were produced in the fast reaction of the electronically excited oxygen atom O(^1^D) with water molecules:

O(^1^D) + H_2_O → 2 OH (1)

The excited oxygen atoms O(^1^D) were generated in ozone photolysis by the UV radiation of the Nd:YAG laser’s fourth harmonic (266 nm, Lotis Tii, model: LS-2137U, Minsk, Belarus). To detect OH in the different excited states, a DFB laser set was used. DFB laser diodes are semiconductor laser diodes, in which a periodic structure is formed directly in the active medium, playing the role of the resonator rear mirror. The laser generates one longitudinal mode; its frequency can be tuned by changing the temperature of a diode. The tuning range of the DFB laser, compared with external resonator semiconductor lasers, is narrow, less than 0.5%. Therefore, a set of DFB lasers is required for working in a wider spectral range. The main advantages of the DFB laser diodes are ease of use and stability compared to the lasers with an external resonator.

An SBF-C43S2 diode laser [22] (tuning range of 6970–7010 cm^−1^) was used for OH detection in the ground state. For OH(ν = 1) detection, the SBF-C50S2 diode laser (tuning range of 6610–6650 cm^−1^) was used, while for OH(ν = 2) detection, we utilized an SBF-C59S2 diode laser at 1589 nm with a tuning range of 6277–6317 cm^−1^. Preliminary calibration of the wavelength was carried out using intensive absorption lines of acetylene and hydrogen sulfide.

### 2.2. Cross-Sections of the Transitions

**Figure 2:**
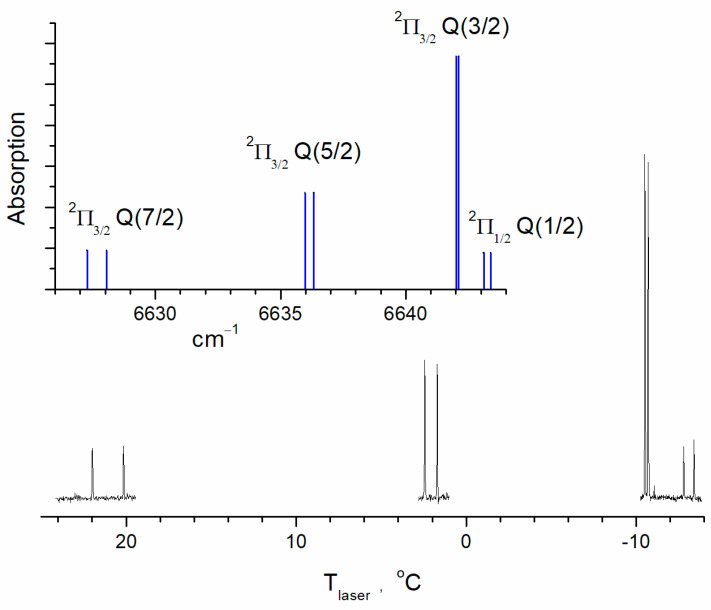
The OH spectrum near Q(3/2) (^2^Π_3/2_ term) of the transition (ν = 3) ← (ν = 1) calculated for a temperature of 296 K. The fragments of the experimental transient absorption spectrum are shown in black and the calculated ones are shown in blue. Concentrations are [H_2_O] = 2.8 × 10^16^ cm^−3^; [He] = 1.9 × 10^17^ cm^−3^; [O_2_] = 10^16^ cm^−3^; and [O_3_] = 5 × 10^14^ cm^−3^. The spectrum was recorded by slowly tuning the laser temperature.

The line intensities of OH (or Einstein A coefficients) in the databases are the calculated values [23,24]. Considering the accuracy of the intensities for the aforementioned lines, the HITRAN2020 database gives an intensity uncertainty of 10% to 20%. The cross-sections of the lines were calculated using the SPECTRA program [25] based on the international spectroscopic databases. The Q(3/2) cross-sections of the transitions ν = 2 ← 0, ν = 3 ← 1, and ν = 4 ← 2 were determined under equilibrium and low-pressure (0.01 bar) conditions at a temperature of T = 298 K. The collisional absorption line width under these conditions was less than 10^−3^ cm^−1^, while the Doppler width was 0.018 cm^−1^. Therefore, the possible uncertainty in the parameters of collisional broadening was insignificant. The cross-sections of vibrationally excited OH radicals were then recalculated by taking into account the population of OH(ν = 1) and OH(ν = 2) levels under equilibrium conditions. The values σ_0_ = 5.74 × 10^−19^ cm^−2^, σ_1_ = 1.72 × 10^−18^ cm^−2^, and σ_2_ = 3.34 × 10^−18^ cm^−2^ were obtained for transitions ν = 2 ← 0, ν = 3 ← 1, and ν = 4 ← 2, respectively. The equilibrium distribution over the rotational states for vibrationally excited molecules was assumed. Also, equilibrium populations of the fine structure and the Λ-doublet levels were assumed. The experimentally obtained spectra confirmed this assumption for OH(ν = 1) (Figure 2).

## 3. Results

Figure 2 shows part of the hydroxyl radical spectrum containing the most intense line of the rotational structure of ν = 3 ← 1. For comparison, we show the calculated spectrum for a temperature of 296 K using the SPECTRA program [25]. The Q(3/2) doublet of the ^2^Π_3/2_ term is the most intensive one at 6642 cm^−1^. The Q(5/2) doublet of the same electron term is at 6636 cm^−1^, and at 6627 cm^−1^, there is the Q(7/2) doublet. The spectrum of the ^2^Π_1/2_ term is similarly structured but less intensive. The lower part of the figure shows fragments of the transient absorption spectrum. The spectrum was recorded in the time window of, ca., 4 μs after the photolysis pulse. The laser frequency was tuned by changing the laser temperature. The rate of temperature scanning was 0.001 °C/s. The frequency dependence on temperature was preliminarily determined using the absorption lines of C_2_H_2_. The accuracy of this calibration was 0.05 cm^−^^1^. Within this accuracy, the position of the observed absorption lines coincided with the HITRAN data. The technique for recording transient spectra was described in detail in [26]. The buffer gas (He) pressure was 6 Torr, so the linewidths were close to those caused by the Doppler broadening. The relative line intensities of the observed spectrum correspond to the calculated relative ones, which means that the population distribution along the rotational lines approximately corresponds to the equilibrium. This also refers to the population of the ^2^Π_1/2_ and ^2^Π_3/2_ states. A simple estimate of the rotational relaxation time is several collisions. Under the experimental conditions (Figure 2), the estimated rotational relaxation time is, ca., 0.1 μs, which is significantly shorter than the characteristic time scale of the experimental signals.

Under experimental conditions, the rotational relaxation time is an order of magnitude shorter than the characteristic time of the processes under study, which means equilibrium in rotational states. Therefore, the measurements were performed on the most intense line of the rotational structure Q(3/2) of the ^2^Π_3/2_ term. It is the most magnetically sensitive line, as the molecular g-factors for different states are 0.935 for the ^2^Π_3/2_(J = 3/2) term, 0.485 for the ^2^Π_3/2_(J = 5/2) term, and 0.325 for the ^2^Π_3/2_(J = 7/2) term [27].

The main results on the detection of vibrationally excited hydroxyl radicals by the absorption of DFB diode laser radiation at the transition ν = 3 ← 1 were presented in [26]. In this study, using a DFB diode at 1589 nm, we were able to directly tune to the transition ν = 4 ← 2 and more accurately determine the yields of vibrationally excited OH(ν = 2) radicals.

The absolute concentrations of OH(ν = 2), OH(ν = 1), and OH(ν = 0) were determined from the absorption signals ∆*I*_2_(*t*), ∆*I*_1_(*t*), and ∆*I*_0_(*t*) using linear equations:

$$\Delta I_{2}\left(t\right)/I_{20}=\sigma_{2}\times n_{2}\left(t\right)\times l;$$

$$\Delta I_{1}\left(t\right)/I_{10}=\sigma_{1}\times n_{1}\left(t\right)\times l;$$

$$\Delta I_{0}\left(t\right)/I_{00}=\sigma_{0}\times \left(n_{0}\left(t\right)-n_{2}\left(t\right)\right)\times l,$$

where *l* is the length of the cell (30 cm); $\sigma_{0}$, $\sigma_{1}$, and $\sigma_{2}$ are the cross-sections of the ν = 2 ← 0, ν = 3 ← 1, and ν = 4 ← 2 transitions, respectively; $n_{0}\left(t\right),\;\;n_{1}\left(t\right)$, and $n_{2}\left(t\right)$ are time-dependent absolute concentrations; and $I_{00}$, $I_{10}$, and $I_{20}$ are the initial intensities.

**Figure 3:**
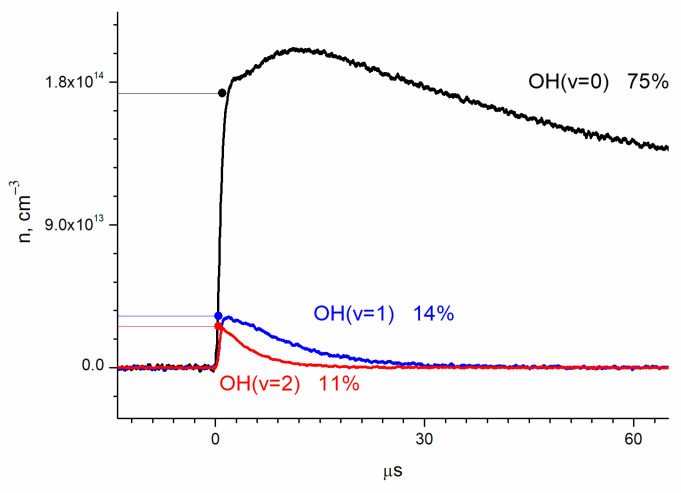
Dynamics of the concentrations of OH vibrational states, determined by absorption at transitions ν = 2 ← 0, ν = 3 ← 1, and ν = 4 ← 2. Concentrations are [O_2_] = 2.8 × 10^16^ cm^−3^; [He] = 1.5 × 10^17^ cm^−3^; [H_2_O] = 6.2 × 10^15^ cm^−3^; and [O_3_] = 1.4 × 10^15^ cm^−3^.

The absolute OH(ν = 2) concentration, obtained from the absorption using the previously stated cross-sections, is shown in Figure 3 together with the results for OH(ν = 1) and OH(ν = 0).

By extrapolation to zero time (laser pulse), the relative yields of OH(ν = 0), OH(ν = 1), and OH(ν = 2) were obtained as 75 ± 10%, 14 ± 2%, and 11 ± 2%, respectively (Figure 3). The errors correspond to the accuracy of the cross-section determination.

In [7], Cheryl et al. studied the nascent product population distribution in the reaction ^16^O(^1^D) + H_2_^18^O → ^16^OH + ^18^OH using the LIF technique. The experiments were carried out under the low-pressure conditions (ca., 0.1 Torr). For ^16^OH, which is formed in the reaction of hydrogen detachment from H_2_O by O(^1^D), a nonequilibrium rotation distribution was observed. For ^18^OH, which is an unreacted remaining fragment of H_2_O, the rotational distribution is approximately equilibrium. The yields of OH were measured separately both for ^16^OH and ^18^OH. The authors claim that the formation of the excited ^18^OH in the second excited vibrational state was not detected. The yields of ^16^OH(ν = 0, 1, 2) were 38%, 41%, and 21%, respectively, while for ^18^OH(ν = 0, 1, 2), they were 91%, 9%, and 0%.

The average distribution for excited states of both ^16^OH and ^18^OH corresponds to the yields of 65%, 25%, and 10%, with which our results were compared. In [7], the yield of OH(ν = 2) is 2.5 times less than the yield of OH(ν = 1), while in our work, they are comparable. A possible reason for the discrepancy is that our experiments, unlike those described in [7], were carried out under conditions of completed rotational relaxation.

The observed relaxation time of the second excited vibrational state of OH is significantly shorter than the relaxation time of the first one.

**Figure 4:**
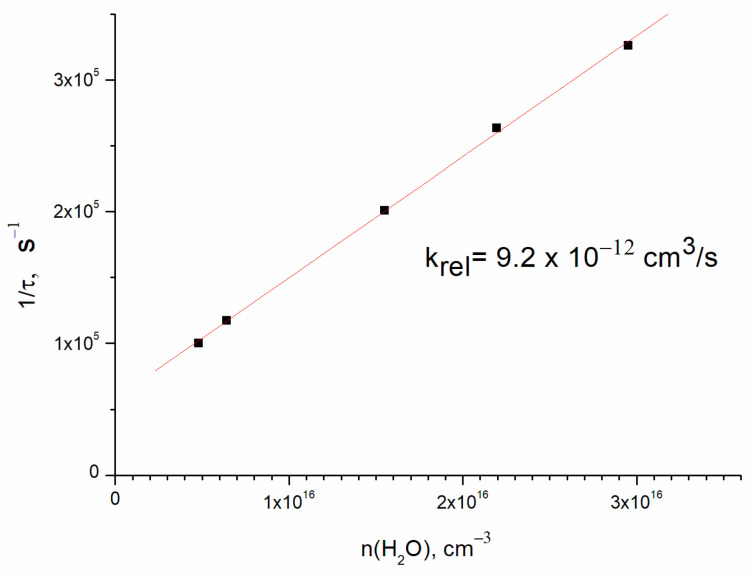
The rate of OH(ν = 1) relaxation vs. H_2_O concentration. Concentrations are [O_2_] = 4.5 × 10^16^ cm^−3^ and [He] = 1.8 × 10^17^ cm^−3^.

Direct OH(ν = 1) detection allowed the determination of the collisional relaxation rate constant. The results of OH(ν = 1) lifetime vs. H_2_O concentration are shown in Figure 4. The slope of the straight line (9.2 ± 2.0) × 10^−12^ cm^3^/s is the rate constant of relaxation of OH(ν = 1) in collision with water molecules, and the finite y axis intercept corresponds to the vibrational relaxation on the buffer gas. The rate constant error corresponds to the uncertainty of water concentration. This result is in agreement with the literature value of 11 × 10^−12^ cm^3^/s [8] for room temperature.

**Figure 5:**
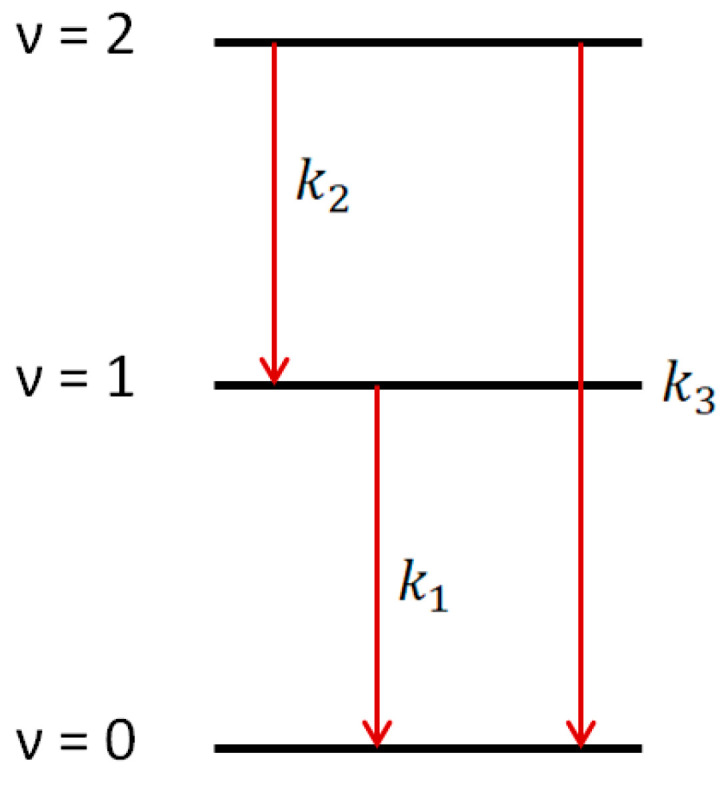
Vibrational relaxation for OH(ν = 1) and OH(ν = 2) channels.

Using 1509 nm and 1589 nm diode lasers for the absorptions on ν = 3 ← 1 and ν = 4 ← 2 transitions, the kinetics of hydroxyl radical excited states OH(ν = 1) and OH(ν = 2) could be accurately traced. This allowed direct observation of the ladder nature of the vibrational relaxation process (Figure 5).

The conclusion about the ladder nature of the vibrational relaxation was obtained based on the solution of the system of differential equations:

(2) $$\left\{\begin{array}{c} \frac{dn_{1}}{dt}=k_{2}n_{2}-k_{1}n_{1};\\ \frac{dn_{2}}{dt}=-\left(k_{2}+k_{3}\right)n_{2}; \end{array}\right.$$

where *n*_1_ and *n*_2_ are concentrations of OH(ν = 1) and OH(ν = 2), respectively, and *k*_1_, *k*_2_, and *k*_3_ are the rates of vibrational relaxation for transitions ν = 1 ← 0, ν = 2 ← 1, and ν = 2 ← 0, respectively (Figure 5). Equation (2) does not take into account the consumption of radicals in reactions with other molecules. The rate of such a process can be estimated from the lifetime of the radical in the ground state. It is about 150 microseconds (Figure 3), which is much longer than the vibrational relaxation time.

From system (2), the time-dependent expressions for the concentrations of OH(ν = 1) and OH(ν = 2) are obtained:

(3) $$n_{1}\left(t\right)=-A_{1}\;exp\left(-\left(k_{2}+k_{3}\right)t\right)+Bexp\left(-k_{1}t\right);$$

(4) $$n_{2}\left(t\right)=A\;exp\left(-\left(k_{2}+k_{3}\right)t\right).$$

At the same time,

(5) $$A_{1}=A\frac{k_{2}}{k_{2}+k_{3}}.$$

**Figure 6:**
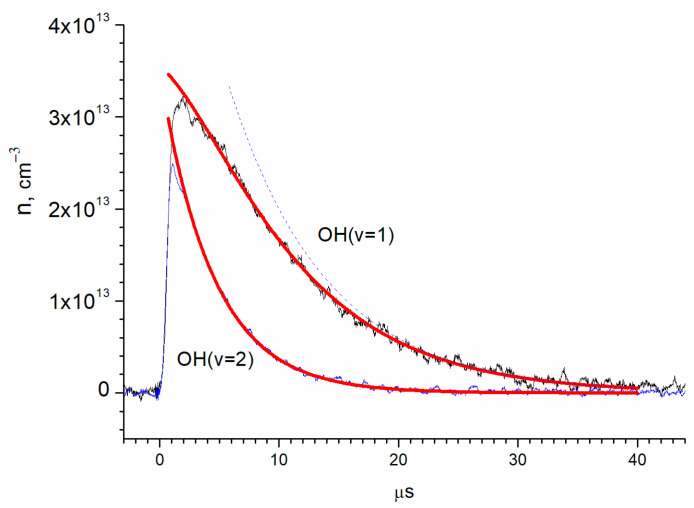
Determination of the vibrational relaxation times of OH(ν = 1) and OH(ν = 2) by exponential approximations. The red lines are exponential approximations according to the (3) and (4). The blue dotted curve shows the contribution of the second term of (3) to the exponential approximation of the OH(ν = 1) kinetic curve.

The parameters *A* and $\left(k_{2}+k_{3}\right)$ were determined by approximating the OH(ν = 2) kinetic curve $n_{2}$(*t*) with an exponential function (Figure 6), where *A* equals (3.50 ± 0.09) × 10^13^ cm^−3^ and ${\left(k_{2}+k_{3}\right)}^{-1}$ equals 4.425 ± 0.013 μs. Then, the OH(ν = 1) kinetic curve was approximated with two exponential functions, while the parameter ($k_{2}+k_{3})$ remained fixed. The parameters $A_{1},\;B,\;\mathrm{and}\;k_{1}$ were determined through $n_{1}$(*t*) approximation, in which *A*_1_ equals (3.20 ± 0.10) × 10^13^ cm^−3^, *B* equals (6.75 ± 0.081) × 10^13^ cm^−3^, and *k*_1_^−1^ equals 8.21 ± 0.04 μs.

Based on the determined values of the parameters, from (5), the contribution of *k*_2_ to the total rate of OH(ν = 2) removal is obtained:

(6) $$\frac{k_{2}}{k_{2}+k_{3}}=0.914\pm 0.046$$

The ratio (6) defines the contribution of the ladder relaxation, which is >90%.

## 4. Conclusions

The time-resolved detection of vibrationally excited hydroxyl radicals by absorption on the overtone transitions ν = 3 ← 1 and ν = 4 ← 2 was experimentally demonstrated. The approach appears to be a simple and quite sensitive method for studying the reactions of vibrationally excited OH. The measured yields of vibrationally excited radicals OH(ν = 0), OH(ν = 1), and OH(ν = 2) in the reaction O(^1^D) + H_2_O are 75%, 14%, and 11%, respectively. The ladder nature of the process of the vibrational relaxation of excited hydroxyl radicals was experimentally confirmed for OH(ν = 2). The Q(3/2) line, on which the measurements were performed, is the most magnetically sensitive; therefore, the increase in the sensitivity of the method using the effect of polarization plane rotation in a magnetic field (Faraday Rotational Spectroscopy technique) is planned [28].

## Data Availability

The raw data supporting the conclusions of this article will be made available by the authors on request.
